# Does signed prediction error drive declarative memory? Evidence from variable choice paradigms

**DOI:** 10.3758/s13421-026-01864-8

**Published:** 2026-03-17

**Authors:** Kshipra Gurunandan, Andrea Greve, Ella Wilmot, Richard N. Henson

**Affiliations:** 1 Medical Research Council Cognition and Brain Sciences Unit, University of Cambridge, 15 Chaucer Road, Cambridge, CB2 7EF UK; 2 Department of Psychology, University of Cambridge, Cambridge, UK; 3 Department of Psychiatry, University of Cambridge, Cambridge, UK

**Keywords:** Episodic memory, Prediction error, One-shot learning, Associative memory, Word pair learning

## Abstract

Prediction error (PE) is the discrepancy between predictions and new information. For a binary reward outcome, PE may be signed (positive if the outcome was better than predicted and negative if the outcome was worse than predicted) or unsigned (absolute value of “surprise”). Using a “variable choice” paradigm, De Loof et al. (*PLOS ONE*, *131,* Article e0189212, 2018) examined the role of PE in one-shot learning of unknown translations of known words and showed that associative memory for the translation was greater when (financial) reward was more unexpected and lesser when an expected reward was not received (i.e., signed PE); an effect that they replicated in several subsequent studies. However, other work on PE in declarative memory has assumed that memory is greater when an outcome is more unexpected, without any explicit reward (i.e., unsigned PE). We replicated De Loof et al.’s paradigm with and without financial reward, and found that memory was explained slightly better by unsigned PE (Experiments 1A–1B). However, we also identified a potential confound in the paradigm that could explain the results without any role of PE, as confirmed by simulations. We therefore designed a modified version of the paradigm that circumvents this confound (Experiment 2). Results were inconsistent with the PE account. We conclude that variable choice paradigms may not be well-suited to investigate the role of PE in one-shot declarative learning, and that the purported role of signed PE in declarative memory requires further investigation.

**Supplementary Information:**

The online version contains supplementary material available at 10.3758/s13421-026-01864-8.

## Introduction

Reward and prediction are central concepts in theories of learning and memory. Reward is generally agreed to enhance learning, with better memory for rewarded stimuli (Adcock et al., 2006; Wittmann et al., 2005), and over repeated experiences, learning is thought to depend on reward prediction error (RPE; i.e., the difference between the actual and predicted reward on each trial; e.g., Wagner & Rescorla, 1972). This is the basis of the “delta rule” used for back-propagation learning in artificial neural networks (Rumelhart et al., 1986), and there is neurophysiological evidence of neurons coding this RPE (Schultz, 2016). In these conceptions, learning depends on the *sign* of the RPE: with the associative strength between a cue and a reward increasing on trials when a reward occurs that is not highly predicted, but decreasing when a predicted reward does not occur.

While there is still a debate on the exact mechanism(s) by which prediction error improves learning across multiple trials (e.g., in terms of modulating cue salience/attention; Le Pelley & McLaren, 2003; Pearce & Hall, 1980), more recent studies have investigated the role of prediction error in “one-shot” (single-trial) associative memory. For example, in the “Variable Choice Paradigm” (VCP) introduced by De Loof et al. (2018), participants predict one stimulus on the basis of another, which is followed by a financial reward if their prediction is correct. Participants’ later memory for the stimulus–stimulus association was reported to be a linear function of signed RPE (an effect replicated in several subsequent studies; e.g., Calderon et al., 2021; Ergo et al., 2019, 2021). The stimuli being associated were a known and an unknown word, analogous to learning the translation of a word in a foreign language. Importantly, these studies were the first to show that signed prediction error could drive one-shot episodic memory for paired associates, setting the path for potential applications such as optimising learning of new vocabulary.

However, other studies of people’s ability to explicitly remember new stimulus–stimulus associations on the basis of a single trial have assumed that an important driver of such declarative memory is the *unsigned* prediction error (Ergo et al., 2020). These assumptions are often based on notions of “surprise,” such as the divergence between probability distributions representing the expectation (prior) and the sensory evidence (likelihood) within a Bayesian framework (e.g., the PIMMS framework of Henson & Gagnepain, 2010). This divergence is always positive, just capturing the degree to which an outcome deviates from expectations. However, most of the studies supporting unsigned prediction error have not used an explicit reward; they typically train an expectation of one neutral stimulus to be paired with another, to varying degrees, and then violate that expectation to test how well the violating event is remembered (e.g., Greve et al., 2017).^1^ Thus one possibility is that the sign of prediction error matters only in situations of explicit reward; another possibility is that both signed and unsigned prediction error contribute to memory, in proportions that might depend on reward, but also other task parameters (Ergo et al., 2020; Rouhani et al., 2023.

Much of the evidence for signed versus unsigned prediction error effects come from reinforcement learning paradigms. In these paradigms, a stimulus is presented, which triggers an action, after which a reward is provided, which gradually alters the stimulus–action contingency across multiple trials (e.g., Jang et al., 2019; Ortiz-Tudela et al., 2018, 2023; Rouhani & Niv, 2021; Rouhani et al., 2018). In this context, RPE is the difference between the actual and expected reward,^2^ which is typically manipulated by varying magnitude and/or probability of financial reward. To facilitate testing of episodic memory in the same paradigm, the reward is accompanied by an unrelated trial-unique image, and episodic memory for this item is subsequently tested. For comparison, in paired associate paradigms, a first stimulus is presented that predicts an associated stimulus, followed by presentation of a second stimulus, and subsequently, episodic memory for these stimulus–stimulus associations is tested (e.g., Brod et al., 2022; Greve et al., 2017). There is typically no action or explicit reward, and prediction error is proportional to the difference between the actual and expected stimuli. The VCP combines features of both types of paradigms: financial reward, as in reinforcement learning paradigms, along with trial-specific predictions, as in paired associate paradigms. Reinforcement learning paradigms have shown that signed RPE drives learning across trials, while episodic memory for unrelated stimuli is driven by unsigned RPE (e.g., Rouhani & Niv, 2021; Rouhani et al., 2018; though Jang et al., 2019, failed to find this latter effect). Consistent with these results, paired associate paradigms have found an effect of unsigned prediction error on one-shot episodic memory. Interestingly, however, the VCP showed evidence for an effect of signed RPE even in one-shot associative memory.

In the present study, we set out to compare models of signed versus unsigned prediction error (SPE vs UPE) in their ability to fit declarative memory for word pairs using the VCP. We first repeated the design of De Loof et al. (2018) without financial reward, and found the UPE model to be slightly better than the SPE model (Experiment 1A). We then followed up with a closer replication of the original study, complete with financial reward, but still found the UPE model to be better (Experiment 1B). However, in the course of running these experiments, we identified an alternative explanation for the results from this paradigm that does not appeal to either UPE or SPE. This was based on participants’ reports of how they approached the VCP task (resonating with our own impressions when performing it), which we implemented in a simple “multiple trace” model. Simulations of this model showed that it can explain the pattern of results across all previous VCP studies. Using this model, we designed a final experiment that controlled for the potential bias arising from multiple memory traces, and which now showed no evidence of either UPE or SPE (Experiment 2). We conclude that the VCP is not ideal for studying the role of prediction error in associative memory.

## Experiment 1A

This experiment was preregistered on OSF(https://osf.io/eubzf).

### Methods

#### Participants

Sample size was determined by power analysis on the primary effect of interest—namely, that declarative memory performance improves linearly with SPE. De Loof et al. (2018) reported χ^2^ = 27.4 for this linear effect, which was converted via the formula φ = √(χ^2^/*N*) to give an effect size of φ = 0.86. Using a χ^2^ goodness of fit test, *N* = 15 is sufficient to replicate an effect size of 0.86 with more than 90% probability (with alpha = 0.05). To err on the side of caution, we halved this effect size, and arrived at a sample size of *N* = 57, which we rounded up to 60.

Of the 60 participants tested, one half were recruited in Cambridge, UK (primarily undergraduate students at the University of Cambridge), and tested in person (*N* = 30, 13 men, median age 20 years ranging from 18–23). The other half were recruited via Prolific (https://prolific.com/), a web-based, international crowd-sourcing platform, and performed the experiment online (*N* = 24, 16 men, median age 30.5 years ranging from 19–35). An additional six participants from the online group were excluded because their data did not meet the criteria specified in our pre-registration (see below). All participants were required to be aged 18–35 years, have no prior history of psychiatric or neurological conditions (including dyslexia and ADHD), have English as their primary language and no prior exposure to Swahili. For the online group, they also needed an approval rate of at least 98% on Prolific. Prior to beginning the experiment, participants provided informed consent, and were compensated financially for their time. The programme of research was approved by Cambridge Psychological Research Ethics Committee (reference CPREC 2020.018), and all procedures accorded with the Declaration of Helsinki.

#### Materials

Sixty English and 240 Swahili words were provided by the original authors (De Loof et al., 2018), and the stimuli were constructed by randomly pairing each English word with four unique Swahili words. These stimuli were dynamically assigned to different conditions across participants as described below.

#### Procedure

**Fig. 1:**
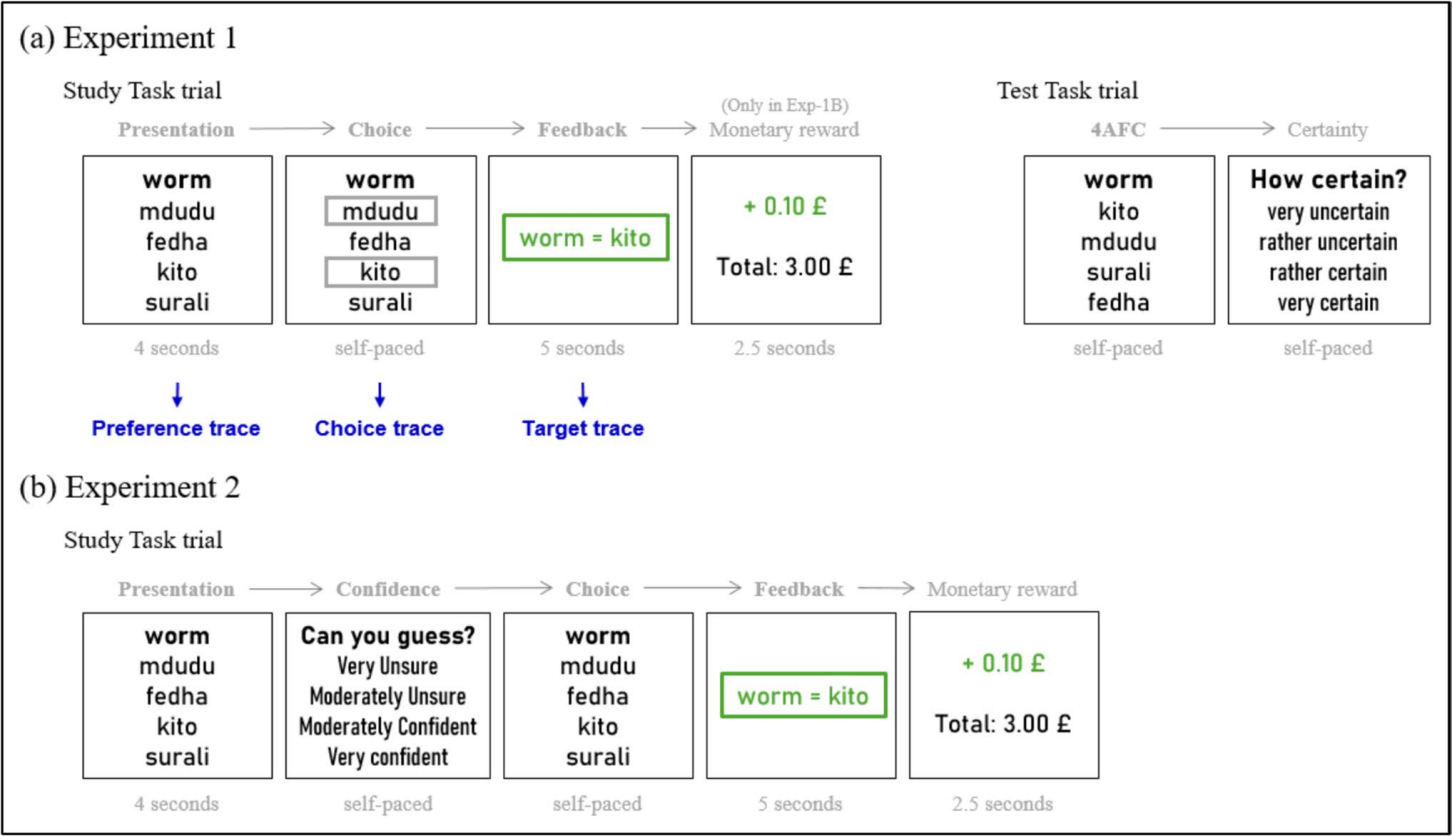
Trial structure for the study and test tasks. **(a)** In Experiment 1A, study task trials unfolded in three steps: on each trial, participants were first presented an English word and four Swahili words; then, one, two, or four of the Swahili words were framed in grey, and participants were instructed to predict the correct translation from the framed options. The correct word pair was then displayed in green if the prediction was correct and in red if the prediction was incorrect, and participants had 5 s to memorise the correct word pair. In Experiment 1B, participants performed the same study task but also received a monetary reward for each correct prediction (and no reward for wrong predictions). In the test task trials, participants once again saw an English word with four Swahili words and had to choose the correct translation (according to the study task feedback) and rate their certainty. The blue text illustrates the “memory trace” for each step of the study task in our multiple-trace account of the results. **(b)** In Experiment 2, participants performed a modified version of the study task and the same test task as in Experiment 1. On each study task trial, participants were first presented an English word and four Swahili words; they then rated their prediction confidence and were able to choose any of the four words. They then received feedback and monetary reward

The experiment followed a similar procedure as the original experiment (De Loof et al., 2018), but without performance-based financial reward (see Fig. 1a).

**Stage 1—Familiarisation:** Participants were first exposed to all the English and Swahili words that they would encounter in the subsequent stages. The intermixed words appeared one by one on the screen for 2 s, and participants were asked to read each word out loud and press the button “e” whenever they saw an English word. This task also doubled as an attention check: lower than 75% accuracy resulted in automatic termination of the experiment, and participants were compensated for the time spent until this point.

**Stage 2—Study task:** Participants learned 60 arbitrarily associated English-Swahili word pairs with the following procedure. On each trial, participants saw an English word paired with four Swahili words. After 4 s, either one, two, or four Swahili words were framed in grey (at random for each participant), and participants were instructed to “predict” the correct translation from the framed options. Participants were allowed plenty of time as to make their choice.^3^ Once participants made their choice, they received feedback (i.e., the correct word pair was displayed in one of two ways: if the participant choice was correct, the word pair was displayed in green, but if the participant choice was incorrect, the correct word pair was displayed in red). The correct word pair was displayed for 5 s, and participants were instructed to learn this word pair. The “correct” word pairs were determined dynamically for each participant based on their choices and the number of Swahili options, such that regardless of their actual choice, they were always correct at chance level (i.e., 50% in the two-option condition and 25% in the four-option condition). So in the one-option condition, the (randomly) framed word was the correct translation in all trials; in the two-option condition, the participant’s choice was set as the correct translation on 50% of the trials; in the four-option condition, the participant’s choice was set as the correct translation on 25% of the trials; and when participant choice was deemed incorrect, a different framed option was randomly set as the correct translation. Hence the “correct translations” were specific to each participant.

**Fig. 2:**
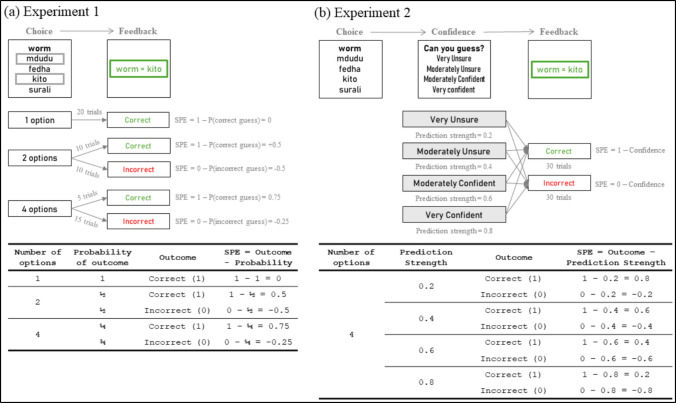
Experimental manipulation and prediction error calculations. **(a)** In Experiment 1, the probability of reward is manipulated by varying the number of options, and SPE is calculated based on the probability of correct/incorrect guessing—that is, 1/number of options (assuming that participants made choices at random). **(b)** In Experiment 2, participants were asked to rate their confidence in their guesses (based on participant feedback of using various strategies to choose options), and SPE was calculated based on these trial-by-trial participant ratings (assuming a scale of 0.2–0.8, omitting the extremes of no prediction (0) and perfect confidence (1)). (Colour figure online)

Here, signed prediction error (SPE) is conceptualised as the probability of guessing correctly (i.e., ¼ in the four-option condition, ½ in the two-option condition, and 1 in the one-option condition) subtracted from the actual outcome (i.e., 1 in the “correct” condition and 0 in the “incorrect” condition; see Fig. 2). In the one-option condition, the outcome is fully expected and the feedback elicits no SPE. The two-option condition implies 50% probability of making a correct response and elicits a positive PE on positive feedback and negative PE on negative feedback. In the four-option condition with 25% probability of a correct guess, a correct response is less expected and thus produces a larger positive PE, whereas an

incorrect response is more expected and therefore elicits a smaller negative PE. Accordingly, the probability of receiving a positive reward decreases with set size (1 for one-option → ½ for two-option → ¼ for four-option), and the SPE varies accordingly.

**Stage 3—Test task:** Participants then performed a four-alternative forced-choice (4AFC) task. On each trial, participants once again saw each English word with four corresponding Swahili options (presented in a different order than at encoding) and had to pick the correct Swahili translation. Participants then rated their certainty on a four-point scale with 1 = “very uncertain” and 4 = “very certain.”

#### Analysis

Data were analysed using R Version 4.2.2 and RStudio 2022.02.3, using code available on OSF (https://osf.io/b48ga). As specified in our preregistration document, we had two exclusion criteria for the data: i) trials with reaction times (RT) below 200 ms; ii) participants with below-chance performance, where chance was determined by permuting their responses 10,000 times, and testing whether actual performance was in the 95^th^ percentile of the null distribution. This led to exclusion of four participants, plus another two who had fewer than 10 trials remaining after the RT cut-off above.

Signed prediction error (SPE) at encoding was calculated for each trial as follows:

$$SPE=Reward- \frac{1}{Number of Options},$$

where *Reward* was 1 in rewarded trials and 0 in unrewarded trials (i.e., correct/incorrect feedback since there was no financial reward in this experiment), and *Number of Options* was the number of Swahili options (one, two, or four) from which participants had to predict the correct translation.

Recognition memory performance was analysed using logistic linear mixed-effects models implemented with the R package *lmerTest*. All predictors were *Z*-scored (as in De Loof et al., 2018), and models were compared using AIC (Akaike information criterion) and BIC (Bayes information criterion).

Based on the SPE model of De Loof et al., we first modelled retrieval performance as follows:

$$Accuracy \sim SPE*Collection+1\left|Stimulus+1\right|Participant,$$

where *Accuracy* was 1 for correct responses and 0 for wrong responses, *Collection* was a binary factor indicating whether the data were collected in person or online, and *Stimulus* (English word) and *Participant* were included in the model as random intercepts.

We compared this SPE model to various models of UPE. The first simply replaced SPE above with the absolute value of UPE = |SPE| (i.e., V-shaped around 0), while the second replaced with UPE = SPE^2^ (i.e., U-shaped around 0). Third, as in our preregistered analysis, we also fit a second-order polynomial expansion with both a linear (SPE) and quadratic (UPE) term:^4^

$$Accuracy \sim {SPE+ SPE}^{2}+ Collection+1\left|Stimulus+1\right|Participant.$$

Finally, we tested the additional model proposed by De Loof et al., which regresses out potential confounds of i) presence/absence of Reward (modelled as a binary effect) and ii) number of options at study (#Options, modelled as a linear effect). Using the definition of PE according to the model with the best AIC and BIC (see Results), this model was as follows:^5^

$$Accuracy \sim PE+Collection+Reward+\#Options+1|Stimulus+1|Participant. 5$$

### Results

**Fig. 3:**
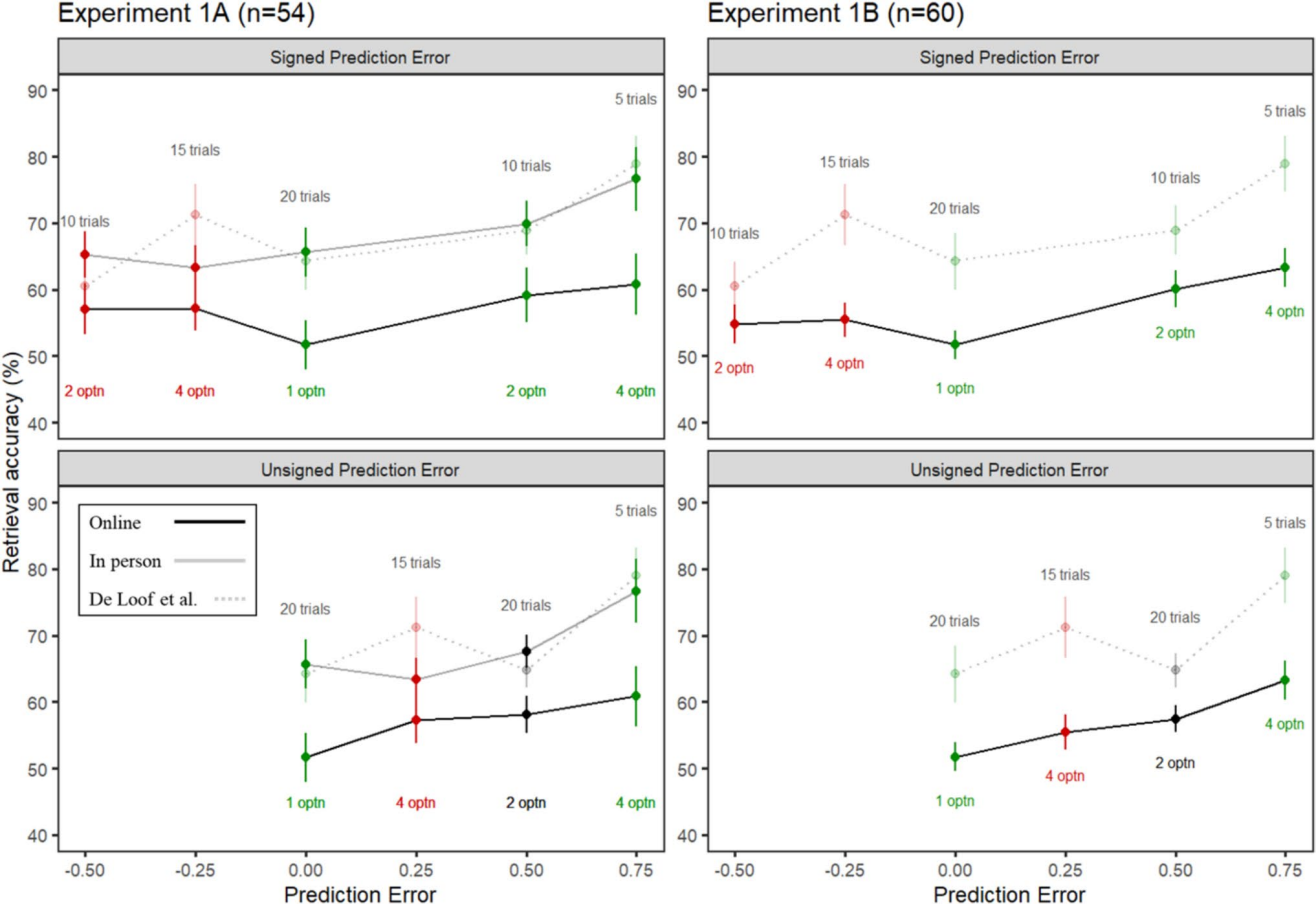
Mean retrieval accuracy in the 4AFC task plotted as a function of signed prediction error (top panels) and unsigned prediction error (bottom panels). Experiment 1A shows separate lines for in-person (black) and online data (grey). The lighter points and dotted line show the corresponding (in-person) results from De Loof et al. (2018; their Experiment 1’s immediate testing group with sample size of *n* = 19). Rewarded trials are plotted in green, unrewarded trials in red, and black indicates averaging over rewarded and unrewarded trials. The corresponding number of options (“optn”) in each condition and the total number of trials are labelled. The error bars show standard error of the mean. (Colour figure online)

All participants had a total of 58–60 valid trials. Overall accuracy in the 4AFC task was 61.89% (in person: *M* = 66.62, *SD* = 15.92; online: *M* = 55.97, *SD* = 13.03). Figure 3a shows accuracy as a function of signed (top left) and unsigned (bottom left) absolute prediction error (solid lines), overlaid with corresponding results from De Loof et al. (their Experiment 1’s immediate-testing group, in dotted lines).

The SPE model replicated the results of De Loof et al., with a significant positive, linear effect of SPE (*z* = 3.05, *p* =.002). Indeed, all models showed a significant positive effect of PE (Table 1). They also all showed a main effect of Collection, with better overall performance in person than online, but this did not interact with PE in any of the models in which the interaction could be estimated (*p* >.133). In addition to this linear effect, the second-order polynomial model showed a significant, positive quadratic component (*z* = 2.44, *p* =.015).

**Table 1:** AIC and BIC values for the five models, with best model shown in bold, and differences from the best model in brackets

Model	Num. Par.	AIC (diff from best)	BIC (diff from best)
***Experiment 1A***			
SPE	5	4093.3 (4.4)	4123.7 (4.4)
UPE = |SPE|	5	4090.6 (1.7)	4121.0 (1.7)
UPE = SPE^2^	5	**4088.9 (0)**	**4119.3 (0)**
SPE + UPE (SPE^2^)	6	4089.3 (0.4)	4125.8 (6.5)
PE + Reward + #Options	7	4091.3 (2.4)	4133.8 (14.5)
***Experiment 1B***			
SPE	4	4711.1 (7.8)	4735.8 (7.7)
UPE = |SPE|	4	**4703.3 (0)**	**4728.1 (0)**
UPE = SPE^2^	4	4704.0 (0.7)	4728.7 (0.6)
SPE + UPE (SPE^2^)	5	4704.4 (1.1)	4735.4 (7.3)
PE + Reward + #Options	6	4703.4 (0.1)	4740.5 (12.4)

Num. Par. = number of parameters. For fair comparison, the term for the interaction with Collection was removed from all models. “PE” in final model refers to definition from best of previous models.

According to both AIC and BIC, the best model was one with UPE = SPE^2^, though the differences between the various UPE models were small (Table 1). By contrast, the original SPE model was significantly worse (in terms of a difference in AIC/BIC > 2; Bevans, 2020).

Finally, using the definition of PE = UPE = SPE^2^, AIC and BIC did not suggest that adding the additional confounds of Reward and #Options improved the winning model (Table 1), and this model still showed a significant main effect of UPE (*z* = 2.57, *p* =.010), demonstrating that the effect of UPE is not a simple linear consequence of the two variables that define it.

#### Interim discussion

Overall, we replicated the basic pattern of results from De Loof et al. (2018) with a larger sample size. However, our analyses indicated that the data were explained slightly better by the squared value of prediction error (i.e., unsigned prediction error). To investigate whether this might have to do with the lack of financial reward in our experiment, we ran the same experiment, this time incorporating financial reward as in the original De Loof et al. study.

## Experiment 1B

Experiment 1B was a close replication of Experiment 1A, but with financial reward. Since our previous results revealed no interaction between method of data collection and the effects of interest, this experiment was conducted entirely online. The preregistration can be found on OSF (https://osf.io/8wtsn).

### Methods

#### Participants

The final dataset consisted of 60 participants (40 men, median age 30 years ranging from 19–35), recruited from the same pool of Prolific users as before (excluding those who had participated in the previous experiment). To avoid any bias, the recruitment materials were identical to those of the previous experiment and did not mention bonus payments. A total of 76 participants completed the experiment, from which 16 were excluded because their data did not meet the criteria specified in our preregistration.

#### Materials

Materials were identical to the previous experiment.

#### Procedure

The only difference from Experiment 1A was in the feedback phase (Fig. 1). In addition to the correct word pair turning green or red, a “ka-ching” sound or the sound of an error buzzer was presented for correct or incorrect trials respectively. After 2.5 s, the screen displayed a reward of either £0.10 for correct trials, or £0 for incorrect trials, as well as their running total reward. Since the proportion of rewarded trials was pre-determined, all participants received a total bonus of £3.50.

#### Analysis

Analyses were identical to the previous experiment (but without the need for a *Collection* factor in the models).

### Results

All participants had a total of 58–60 valid trials. Overall accuracy in the 4AFC task was 55.53% (*SD* = 15.97), which is similar to the online group in Experiment 1A. The top right and bottom right panels of Fig. 3 plot results as a function of signed and unsigned prediction error, respectively.

As in Experiment 1A, we found significant positive linear effects of PE in all three simple models (*p* <.006). The full model with a second-order polynomial also showed a significant positive quadratic component (*z* = 2.94, *p* =.003).

As in Experiment 1A, both AIC and BIC showed that the SPE model fit was significantly worse than the UPE models (Table 1). This time, the model with the absolute value of SPE had slightly better AIC/BIC than that with squared SPE, though again not significantly so (AIC/BIC difference < 2). Neither AIC and BIC suggested that the additional confounds (Reward and #Options) were needed, and this model still showed a significant main effect of UPE = |SPE| (*z* = 3.07, *p* =.002).

#### Interim discussion

The results of Experiment 1B were nearly identical to those of Experiment 1A (and De Loof et al., 2018), suggesting that the addition of financial reward had little effect on the relationship between prediction error and memory. Again, the UPE models fit the data better than the SPE model.

We do not wish to claim too strongly that UPE is a better model than SPE, since De Loof et al. reported the opposite result, where the SPE model fit their data better than the UPE models, and it is possible that there are residual methodological differences between the experiments that affect the data. More important is our observation that, following feedback from participants (and from participating in the experiment ourselves), there might be a potential confound in the paradigm that could explain the pattern of results without any recourse to prediction error, signed or unsigned. Participants often reported looking for linguistic patterns that might predict the correct translation, even though the English and Swahili words were paired completely at random to avoid any such cues (a fact that was unknown to the participants). This suggests that participants’ predictions (and hence prediction errors) might not simply be a function of the number of options, but also depend on their individual prior beliefs (i.e., preferences for the translation). We call this the “multiple-trace” account below, in that there may not only be a memory trace for the correct translation (indicated by feedback), but also other traces, such as the participant’s preferred choice (regardless of whether subsequently correct or not). We formalise this possibility in the next section, and show how it can mimic the effects of SPE.

#### A multiple-trace account

According to this account, before a subset of options are framed during an encoding trial, participants have a preference for one of the four Swahili options as the translation of the English word (e.g., based on morphological comparisons between the two languages). The probability of this preference then being framed depends on the condition: in the one-option condition, there is a 25% chance that the participant’s preferred translation is framed; in the two-option condition, the chance is 50%; and in the four-option condition, there is a 100% certainty that the participant’s preferred translation is framed. If their preference is framed, it is assumed participants will choose their preference; otherwise, they randomly select one of the framed options.

Then during a retrieval trial, when participants are again presented with an English word and four Swahili words, they have a certain chance of remembering the correct translation defined by the feedback they received at encoding (the “Target trace” in Fig. 1). The probability of this correct retrieval is assumed to be independent of condition (i.e., regardless of SPE). However, if they do not remember the correct translation, there is assumed to be a chance that they will remember their original preference (the “Preference trace” in Fig. 1), without remembering whether it was correct or not. This second trace could be episodic (a memory for what they thought at encoding) or semantic (the same preference they would always have about the likely translation, even if they do not remember thinking about that preference at encoding). Either way, assuming they choose this preference at test, then the chance that it will be correct does depend on the condition, as expanded below. For completeness, there could also be a third episodic trace (the “Choice” trace in Fig. 1), capturing the choice that the participant actually made at encoding (which could differ from their preference if their original preference was not framed, and differ from the target trace if this choice was not rewarded). If none of these traces is retrieved, then participants are assumed to guess randomly.

Considering only a dual-trace model for the moment (with Preference and Target traces), to facilitate explanation, consider a test trial where participants do not remember the correct translation and choose their preference instead. Since a proportion of the correct word pairs were determined based on participant choice at study (i.e., 50% of two-option trials and 25% of four-option trials), the chance of their preference being the correct translation depends on the number of options during the study phase (i.e., the probability of their preference being framed), and whether that study trial was rewarded (i.e., if their choice was deemed correct). On the other hand, if their preference was either not framed at study, or was framed but not rewarded when they chose it, then they will always be incorrect if they choose their preference again at retrieval. These possibilities are shown in Table 2.

**Table 2.**
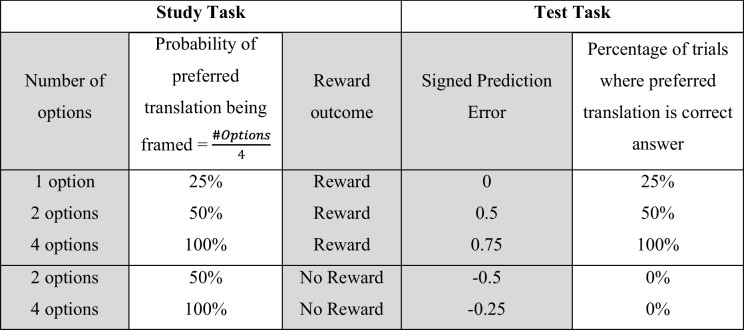
Illustration of predictions of a dual-trace account for relative performance across conditions in Experiment 1

The columns shaded in light grey (first, third and fourth) denote the experimental conditions, and the unshaded columns (second and fifth) show our hypothesised consequences. The first unshaded column denotes the probability of participant preference being available as a consequence of framing 1/2/4 options, and the second unshaded column is either the same as the first (if choice rewarded) or 0 (if choice unrewarded).

From this table, it can already be seen that, even if participants never remembered the correct translation (the target trace was inaccessible), the presence of a second “preference trace” would predict a pattern of memory performance that closely mimics that assumed by SPE, with higher performance in rewarded trials than in unrewarded trials, and linear increase with increasing number of options within the rewarded trials. One difference from the predictions of SPE is that performance would not be expected to differ across unrewarded conditions (i.e., between the −0.5 and −0.25 SPE conditions, and indeed, there was little suggestion of such a difference in Experiments 1A and 1B). Indeed, this dual-trace account predicts both the linear and quadratic components that we found (rather than the purely quadratic component predicted by UPE account), since its predictions are not symmetrical around SPE = 0.

Note that in this account, the SPE-like pattern of results arises primarily from a quirk of the trial structure of the study task, i.e., the inclusion of the presentation stage (Fig. 1). This stage was intended to equate sensory exposure to stimuli across the 1/2/4-option trials, but the unintended consequence was that it allowed participants to potentially have preferred options that they could not choose in the one- and two-option trials, leading to a separate but overlapping “preference trace” in addition to the “choice trace.” On its own, the choice trace only predicts a choice-confirmation bias (Pupillo & Bruckner, 2023; i.e., a main effect of reward and not a linear increase for rewarded trials with the number of options). However, even without a presentation stage, it could still be argued that more options allow greater probability of a preferred option (Ergo et al., 2021), so simply removing the presentation stage would not fully rule out a multiple-traces account in favour of prediction error.

To confirm the utility of this alternative explanation to SPE, we performed simulations and fit the outcomes to previous empirical results. All simulations were performed with R Version 4.2.2 and RStudio 2022.02.3, with code available on OSF (https://osf.io/b48ga/).

#### ***Simulation 1—Original VCP (***De Loof et al., 2018***)***

The full multiple-trace model uses three parameters to simulate performance: the probability *P(target)* that participants remember the correct translation, L the probability *P(preference)* that they remember their preferred option, and the probability *P(choice)* that they remember the choice made at study. The model was fit using the R function “nls” to provide least-squares estimates of the parameters of a nonlinear model.

**Fig. 4:**
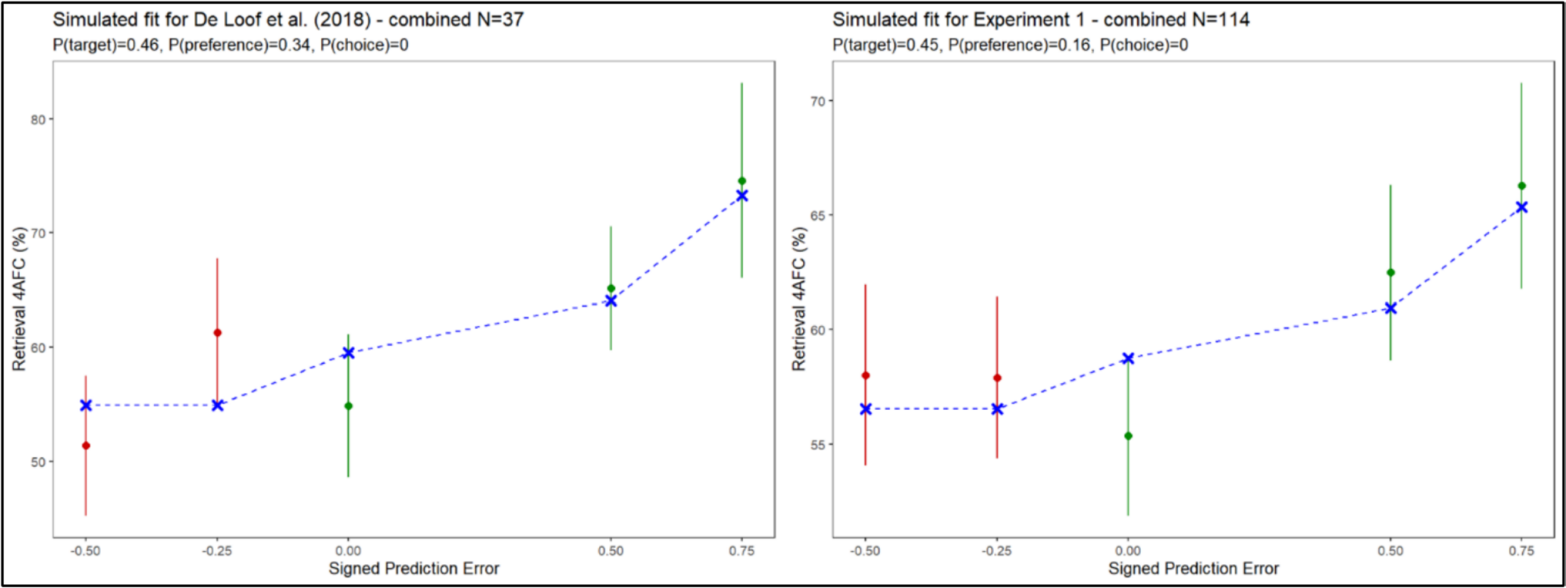
Multiple-trace model (in blue) plotted over data from De Loof et al. (left) and Experiment 1 (right). Error bars are 95% confidence intervals of the empirical data means. (Colour figure online)

Figure 4 shows the model fit to the data from De Loof et al.’s Experiment 1 (immediate and next-day testing) and our Experiment 1 (in-person and online data, and with and without financial reward). These subsets have been combined in Fig. 4 since there were no interactions with delay in the case of De Loof et al., or with method of collection or financial reward across our Experiments 1A and 1B (see Supplementary Fig. S1 for fits split by each condition). In all cases, the model predictions were within the confidence intervals of every data point.

Table 3 shows the parameter estimates for all the experimental subsets. The estimates were reasonably consistent across experiments/subsets. They were also plausible (e.g., the probability of remembering the target was higher than remembering the preference, as would be expected since participants are told to remember the feedback). The third *P(choice)* parameter that we included for completeness was not actually needed to explain the data in the original paradigm, indicating that a dual trace model is good enough to explain the data and the “choice” parameter has very little additional contribution here (more about this in the next section). The mean and standard deviations of parameter estimates after fitting individual participants are shown in Supplementary Table S1.

**Table 3:** Optimised model parameter values for each experimental subset

Experiment	Subset	Model Parameters (triple trace)		
		*P(target)*	*P(preference)*	*P(choice)*
De Loof et al. (2018)	In-person	0.57	0.27	0
	In-person (next day)	0.35	0.38	0
Experiment 1A	In-person	0.56	0.28	0
	Online	0.42	0.08	0
Experiment 1B	Online	0.41	0.15	0
Ergo et al. (2021)	In-person	0.44	0.22	0.08

Corresponding model fits for each subset can be seen in Supplementary Materials (Fig. S1).

#### ***Simulation 2—Modified VCP (***Ergo et al., 2021***)***

**Fig. 5:**
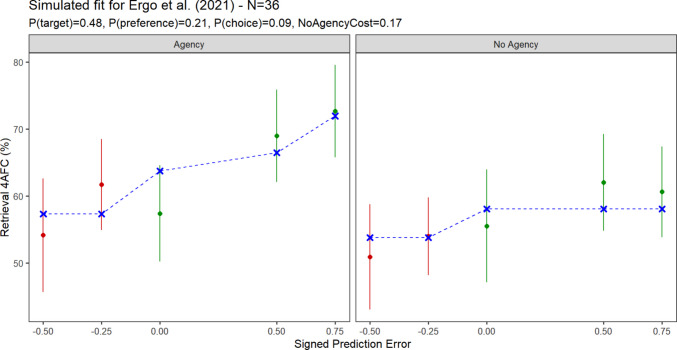
Illustrative fit of the most complex multiple-trace model (in blue) plotted over data from Ergo et al. (2021). Error bars are 95% confidence intervals. All versions of this model have been fitted in Supplementary Materials Fig. S2. (Colour figure online)

In a later study, Ergo et al. (2021) tested whether memory depended on participants’ sense of agency during study. They added an additional factor, such that on one half of the trials, participants were allowed to predict the correct translation as in their previous De Loof et al. (2018) study (“agency” condition), but on the other half, the choice was made for them by the computer (“no agency” condition). The authors reasoned that if participants had preferences for particular translations, this would be irrelevant in the “no agency” condition, which should therefore show no differences between conditions. However, they still found a linear increase across their agency conditions (along with a main effect of Agency), consistent with their SPE account (see Fig. 5 below), and arguing against a pure preference account.

In our account, the “no agency” condition indeed removes the influence of any trace of participants’ preferred option, since the probability of the computer picking that preference is 25% for all levels of SPE. Thus, the *P(preference)* parameter on its own cannot produce any difference (i.e., an effect of Reward or PE) in the “no agency” condition. On the other hand, the memory trace for choice *P(Choice)*, which previously overlapped with participants’ preference in the “agency” condition and thus contributed little in the original paradigm, is now independent of participants’ preference in the “no agency” condition and can play more of a role. This can lead to an effect of Reward in this “no agency” condition, as demonstrated below in Fig. 5.

We fit our triple-trace model to the data from Ergo et al. along with a new parameter to reflect the experimental manipulation of Agency. This additional parameter “*NoAgencyCost*” simply captures the likely decreased attention/motivation in the “no agency” trials compared to the “agency” trials in an additive manner. Note that this additional parameter can produce a main effect of Agency, but it cannot affect SPE conditions differentially (i.e., cannot produce an interaction between Agency and SPE). The model predictions were once again within the confidence intervals of every datapoint (Fig. 5). The parameter estimates for the triple trace model are shown in Table 3, which shows that the *P(choice)* parameter now has a non-zero value and produces a main effect of reward (greater memory for zero and positive SPE conditions) in the “no agency” condition.

However, formal comparison of different versions of the multiple-trace model showed that the dual-trace model had better AIC and BIC (without *NoAgencyCost*: AIC = 3205.8, BIC = 3238.5; with *NoAgencyCost*: AIC = 3208.3, BIC = 3251.9) than the triple-trace model (without *NoAgencyCost*: AIC = 3255.0, BIC = 3298.6; with *NoAgencyCost*: AIC = 3234.2, BIC = 3288.6). The simplest dual-trace model’s predictions also lay within the confidence intervals of all 10 datapoints, even though this model can only produce a flat line in the “no agency” condition, indicating either that these data can be explained by the multiple-traces account or that any linear effect of SPE is very small.

Note that the multiple-trace model predicts a greater linear slope in the “agency” than in the “no agency” condition, since only the former can be affected by participant preference. However, this difference in slopes is dependent on the strength of the preference, and may or may not be statistically significant. Consistent with this prediction, Ergo et al. reported a numerical difference in the predicted direction, and their Bayes Factor indicated no statistical difference. Supplementary Fig. S3 shows linear fits of simulated and actual data, and we see that the SPE and multiple-trace models have very similar slopes and are not meaningfully distinguishable.

Given the plausibility of both the SPE and multiple-trace accounts in all previous datasets, we propose directly testing them in a new experiment where they predict different patterns of results.

#### Simulation 3—Proposed variation on paradigm

In previous experiments, it was difficult to differentiate between the PE and multiple-trace accounts since they predicted the same qualitative pattern of results. We therefore propose a variation on the paradigm where instead of manipulating prediction error through the number of options, we allowed participants to pick any of the four options and rate the confidence in their choice. In this way, participants were able to pick their “preferred option” in every study trial, and we can calculate prediction error in terms of the difference between whether their choice was rewarded and the confidence of their prediction. Thus, PE accounts would still predict a positive, linear effect of SPE, or a V/U-shaped effect of UPE.

**Fig. 6:**
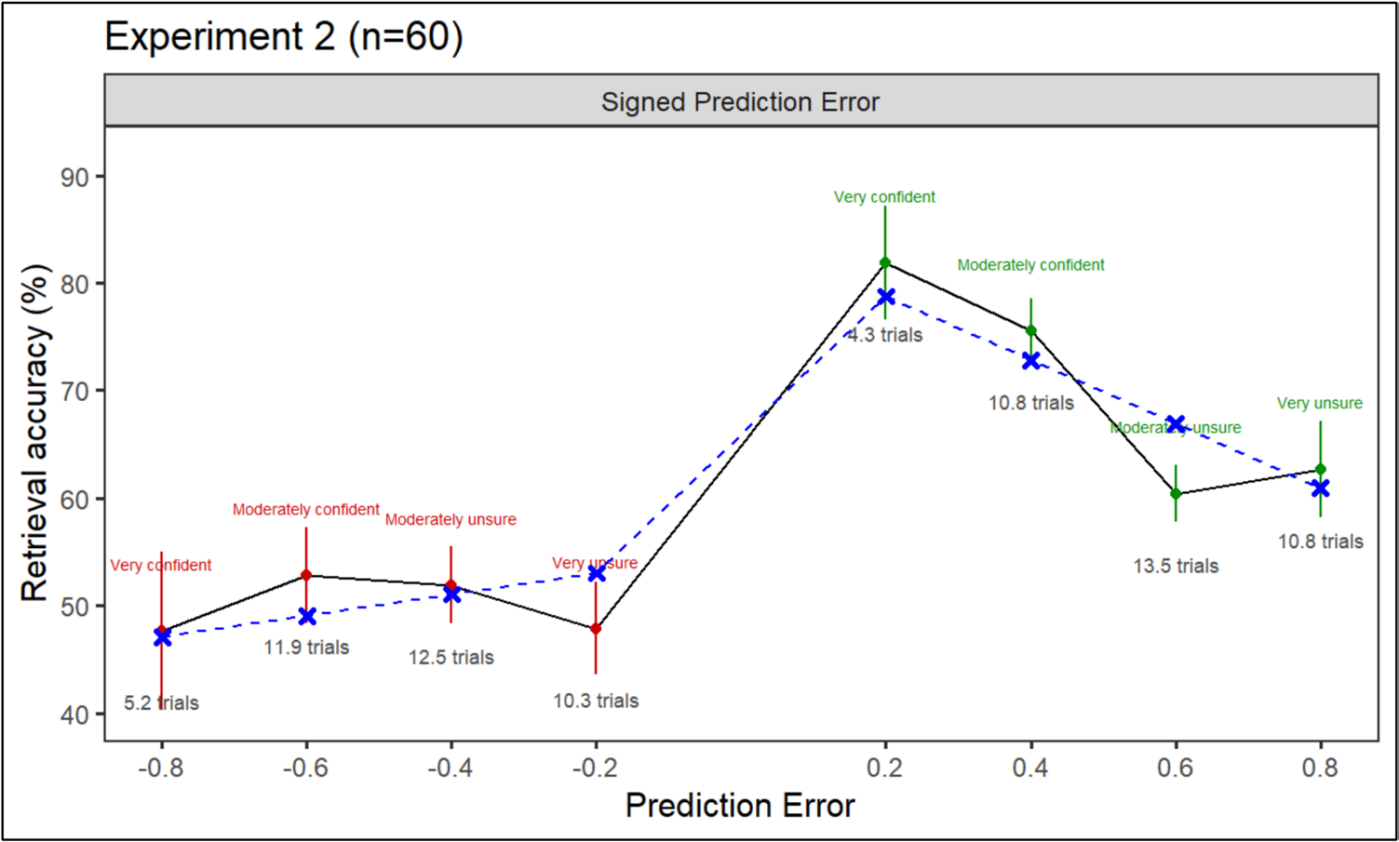
Mean retrieval accuracy in the 4AFC task plotted as a function of signed prediction error. Rewarded trials are plotted in green and unrewarded trials in red, and the corresponding prediction confidence levels and the average number of trials are labelled. The error bars show standard error of the mean. Dual-trace model predictions have been plotted over the data in blue. (Colour figure online)

However, the multiple-trace account now makes very different predictions for memory performance. Firstly, the fact that participants can choose their preference means the preference and choice traces are identical, so the data can be fitted with a dual-trace model with just *P(target)* and *P(preference)*. Secondly, we add the (intuitive) assumption that higher confidence increases *P(preference)*, i.e., more confident preferences are more likely to be retrieved at test (if the target trace is not retrieved). Because rewarded trials mean that the preference happened to be correct, non-zero *P(preference)* values will lead to better memory performance. Unrewarded trials on the other hand mean that the preference was incorrect, so non-zero *P(preference)* values will lead to worse memory (i.e., lead to selection of the wrong choice at test). Thus, the first prediction of this dual-trace model is a main effect of reward, with higher memory for positive SPE (rewarded) conditions than for negative SPE (unrewarded) conditions. Second, because higher confidence leads to larger values of *P(preference)*, the dual-trace model predicts different effects on memory depending on the presence or absence of reward. In the presence of reward (positive SPEs), memory decreases with SPE since stronger positive SPEs are linked to lower confidence responses that were unexpectedly rewarded. Conversely, in the absence of reward (negative SPEs), higher confidence in their preferred translation makes correct retrieval of the actual translation less likely. Thus, the dual-trace model predicts an inverted-V pattern, along with main effect of reward, which is the opposite of UPE, and this is illustrated through the simulated fit in Fig. 6.

#### Interim discussion

Via simulation, we showed that it was possible to reproduce results seen in previous VCP experiments using a simple memory model that assumes only that people have multiple memory traces from different stages of a VCP study trial (their initial preference, the choice they/a computer made, and the correct choice during feedback), and that at test, failures to retrieve the trace corresponding to the correct choice can result in retrieval of one of the other traces guiding the participant’s choice. It turns out that the critical feature of the VCP—the number of choices offered at study (from which different degrees of SPE are assumed to be induced)—also modulates the impact of these other memory traces on performance at test in a manner very similar to what would be expected by SPE, yet without having to appeal to any form of PE.

Our alternative account is not only compatible with findings from the original VCP (De Loof et al., 2018), but also subsequent studies using variants of this paradigm, e.g., with an additional factor of agency (Ergo et al., 2021). Given the compatibility of previous results with both accounts, it is critical to empirically test the two accounts in a paradigm where they make different predictions.

We hence proposed a new paradigm, where we remove the key feature of VCP – the number of options allowed at study—and replace it with participants’ judgment of the confidence of their prediction on each trial. Simulations confirmed that the multiple-trace model now makes different predictions to any type of SPE or UPE model, and we therefore ran this paradigm in Experiment 2.

## Experiment 2

Experiment 2 tests the idea that confidence in the *a priori* preference contributes to the pattern of memory performance in the VCP, rather than prediction error. We made a simple change to the study task, where instead of assuming confidence in predictions was based on the number of framed options, we allowed participants to indicate four levels of confidence associated with their selection of each translation. When crossed with presence/absence of financial reward, this produced eight possible levels of SPE (as defined below). This experiment was preregistered on OSF (https://osf.io/nz295).

In addition to having clearly separable predictions for the multiple-trace and prediction error accounts, this paradigm is also better suited than the original VCP to arbitrate between SPE and UPE accounts. In Experiment 1, the two prediction error accounts overlapped on three out of five levels of prediction error (the nonnegative PE values) and diverged on only two levels (the negative PE values), whereas Experiment 2 has four levels where the accounts diverge and four where they overlap, and consequently, the AIC/BIC for the SPE/UPE models should show much larger differences than in Experiment 1.

### Methods

#### Participants

The final dataset consisted of 60 participants recruited on Prolific (40 men, median age 29 years ranging from 18–35). A total of 62 participants completed the experiment, of whom two were removed because their data did not meet a criterion specified in our preregistration.

#### Materials

Materials were the same as in previous experiments.

#### Procedure

The experimental procedure was identical to Experiment 1B apart from the study task (see Fig. 1). Instead of a subset of words being framed, participants were asked to rate the confidence in their selected translation on a 4-point scale from “very unsure” to “very confident.” Participants were carefully instructed to rate their confidence keeping in mind that they were making guesses and that “very confident” did not mean they had to be sure it was the correct translation. To give the impression that predictions were working above chance level (25%), 50% of responses were chosen at random to be rewarded as the correct translation. The rest of the experiment proceeded as previously described.

#### Analysis

Signed prediction error (SPE) at encoding was calculated for each trial using the formula:

SPE=Reward-Confidence,

where confidence was converted from the 4-point scale to a range of 0.2–0.8 (i.e., assuming participants never experienced the extremes of perfect confidence or perfect uncertainty, though this scaling does not matter for the pattern of results), resulting in SPE values between −0.8 and +0.8 in steps of 0.2 (excluding 0).

We first used the following mixed effects logistic model

$$Accuracy \sim Confidence*Reward+1\left|Stimulus+1\right|Participant$$

to explore basic effects of Confidence and Reward. More importantly, we then used “nls” to fit the trial-averaged means for the eight conditions, to see whether AIC/BIC favoured the SPE, UPE or dual-trace model.

### Results

All participants had 60 valid trials. Overall accuracy in the 4AFC task was 57.81% (*SD* = 16.76). The pattern of memory performance as a function of the eight levels of SPE resembled the inverted-V pattern (plus main effect of reward) expected from the dual-trace model, rather than the linear pattern predicted by SPE or the V/U-shaped pattern predicted by UPE (Fig. 6).

This was confirmed by the mixed-effects model, which revealed a significant interaction between Confidence and Reward (*z* = 1.97, *p* =.049), reflecting a more negative effect of confidence level for rewarded than unrewarded trials, as predicted by the dual-trace model, but not the SPE/UPE models. The relative AIC/BIC values for each model are shown in Table 4, where the dual-trace model was best. The optimised parameter values for the dual-trace model were *P(target)* = 0.40 and *P(preference)* = 0.66. Interestingly, the *P(preference)* parameter was now larger than the *P(target)* parameter, probably reflecting the greater attention paid to the predicted translation owing to the requirement to indicate confidence. Adding an effect of UPE to the model showed that it may have some potential contribution to performance (model with |SPE|: *P(target)* = 0.36, *P(preference)* = 0.67, *UPE effect* = 0.2; model with SPE^2^: *P(target)* = 0.38, *P(preference)* = 0.67, *UPE effect* = 0.16), but AIC/BIC did not suggest that this was needed.

**Table 4:** AIC and BIC values for the models fit to data from Experiment 2, with best model shown in bold, and differences from the best model in brackets

Model	Num. Par.	AIC (diff from best)	BIC (diff from best)
SPE	2	2908.0 (53.9)	2872.5 (53.9)
UPE = |SPE|	2	2987.4 (133.3)	2951.9 (133.3)
UPE = SPE^2^	2	2986.8 (132.6)	2951.2 (132.6)
Dual-trace Model	3	**2854.2 (0)**	**2818.6 (0)**
Dual-trace + |SPE|	4	2933.3 (79.2)	2885.9 (67.3)
Dual-trace + SPE^2^	4	2933.0 (78.8)	2885.6 (67)

Num. Par. = number of parameters. Note that all models in this table were fitted with “nls” to make them fully comparable, so there were no random intercepts and hence two fewer parameters than in Table 1**.**

### Interim discussion

The results of Experiment 2 provided clear evidence against the prediction error account in the variable choice paradigm. In all previous versions of the VCP, the prediction error and multiple-traces accounts predicted similar patterns of results, making it difficult to refute either account. However, Experiment 2 had clearly separable predictions, and the results suggest that previous findings from the variable choice paradigm are unlikely to be attributable to prediction error.

Experiment 2 had the same goals and overall task structure as Experiment 1, and the two experiments differed only in their manipulation of prediction error. The original VCP made the assumption that participants were making random choices based on the number of options, and prediction error was calculated accordingly, with fixed values for all participants. However, the participants in Experiment 1 reported using various strategies to make their choices, from word length and syllable overlap to linguistic patterns. Thus, while participants were no doubt influenced by the number of options, other factors like individual preferences also contributed. Since participants did not know that the stimuli were chosen at random, and were treating the task as a language learning task, it is not inconceivable that any perceived linguistic patterns (i.e., preference) overshadowed the pure statistical probability determined by the number-of-options. In Experiment 2, we removed the number-of-options manipulation, and instead asked participants to rate the strength of their predictions, giving us a trial-by-trial measure of each participant’s preferences. While participants were unaware that the correct answer was manipulated to produce a success rate of 50%, they were told to expect a similar success rate given the “difficulty” of the task. As might be expected by this difficulty, there were relatively few “very confident” responses. Nonetheless, the resulting distribution of ratings, as well as the clear association between confidence and subsequent memory accuracy, indicate that these instructions worked well. Indeed, Experiment 2 showed a stronger effect of prediction strength than Experiment 1 (i.e., the slope of the green points in the corresponding plots).

Overall, since the goal of this task was exactly the same as the original VCP (i.e., selecting one word as the likely translation), and since it provided a purer measure of participant predictions, Experiment 2 should have replicated the effect of prediction error that has been claimed in previous VCP experiments, however, this was not the case.

### Discussion

Across three experiments, we compared signed versus unsigned prediction errors in their ability to explain declarative memory for paired word associates. Using a paradigm that had previously claimed evidence for the signed prediction error (SPE) account (De Loof et al., 2018), we largely replicated their results, but found that unsigned prediction error (UPE) fit the data better, regardless of the presence or absence of financial reward (Experiment 1a and 1b). However, we then used simulations to show that any results from this paradigm and its variants were best explained by a simple, “multiple-trace” model that assumes a memory trace for each stage of the study trials, without appealing to prediction error. When directly pitting this model against prediction error accounts in Experiment 2, we found the pattern of results that was predicted by the model, but that could not be explained by prediction error, signed or unsigned.

Prediction error is often taken for granted as a driver of episodic memory, but direct behavioural evidence for this is sparse. De Loof et al. (2018) were the first to claim evidence for SPE in one-shot, declarative memory for paired associates, which they replicated in subsequent studies using variants of the same paradigm (e.g., Calderon et al., 2021; Ergo et al., 2021). On the other hand, we have previously claimed evidence for the UPE account, with improved memory for unexpected paired associates (Greve et al., 2017) or unexpected numerical facts (Brod et al., 2022). Further support for the UPE account comes primarily from reinforcement learning studies that probed episodic memory for items (though not stimulus–stimulus associations), and found that UPE enhanced item memory (Liu et al., 2025; Rouhani et al., 2018, 2023; Rouhani & Niv, 2021). However, other studies have failed to support either account for item memory (Jang et al., 2019; Ortiz-Tudela et al., 2018, 2023).

Do the present results question previous claims that prediction error drives one-shot associative memory, including our own work? The results of the current study do question such theories, but there are at least two important differences between the present VCP and the paired-associate paradigm we used previously (Greve et al., 2017). For one, the choice of foils in the forced choice tests of Greve et al. (2017) allowed little scope for the multiple traces considered here to bias memory performance. For example, in Greve et al.’s Experiment 2, predictions were trained by learning pairings such as A–B and C–D, after which new trials occurred with A–X or C–Y. In subsequent test trials, participants were asked to remember whether A was paired with X or Y. Because the choices offered at test did not include any stimuli from the training phase (i.e., B and D were never offered), there is little opportunity for alternative memory traces to affect performance (i.e., little scope for proactive interference). This is unlike the present VCP, where traces in which A was assumed to pair with B (even though feedback showed it paired with D instead) can interfere with the forced choice at test for whether A should pair with B or D (as formalised by the multiple trace models described here). In other words, the present multiple trace models would not affect the Greve et al. results, where only one trace can really affect performance. Nonetheless, even though the Greve et al. paradigms do not suffer from the same problems as the VCP, they would still predict an additional (residual) contribution of unsigned prediction error to the results of the present Experiment 2, for which we found limited evidence. Future studies with a different paradigm may reveal such a contribution, for example by removing the opportunity for multiple traces to influence test performance.

The second key distinction is that in Greve et al. (2017), participant predictions were always content-driven (i.e., based on previously studied or experienced material). In the VCP, though the predictions are ostensibly about the content, the expectations themselves emerge from the probabilistic knowledge of the overall likelihood of being correct or incorrect given various choice options. Thus, the expectation violations in the VCP are not inherently tied to the material itself, and as such, any learning from expectation violations may not necessarily update the memory of that material. Furthermore, the assumption that prediction errors occur as expected in every VCP trial may not be true, and instead, expectations may only emerge after experiencing multiple trials with different choices and different feedback (e.g., Pupillo et al., 2023). For example, if a participant selects a random word and gets it wrong, they may simply accept that this was a random occurrence and attribute it to bad luck, rather than experiencing a strong PE. But if they were to fail more *often* than expected, they might experience a violation of their expectations and eventually update their prior belief about the expected success rate within the experiment. Thus again, learning in this paradigm may be driven by probabilistic beliefs that shape the predictions, rather than the material itself.

Overall, it is clear that various factors contribute to episodic memory, and the benefits of prediction errors are not as straightforward as previously conceptualised. It is particularly complicated to interpret results from a paradigm like the VCP that combines features of reward prediction paradigms and content-driven prediction paradigms, and we urge caution in interpreting results from it. A promising way forward may be to test the differences between reward prediction and content-driven predictions in the same experiment, and whether these might map onto distinct types of learning from different signals, including possible signed and unsigned prediction errors.

In summary, the current study originally set out to compare the effect of signed and unsigned prediction error in declarative memory, using a paradigm that had previously shown evidence for the SPE account. Though we were also able to replicate their results, simulations of our alternative model followed by empirical results suggest that it is unlikely that these results reflect prediction error at all. Given that, to the best of our knowledge, the only evidence for an effect of SPE on one-shot, declarative memory for stimulus–stimulus associations comes from the VCP, future work with different paradigms is necessary to shed light on whether SPE plays a role in one-shot declarative learning.

## Supplementary Information

Below is the link to the electronic supplementary material.

Supplementary file1 (DOCX 125 KB)

## Data Availability

Data and materials are available on OSF (https://osf.io/b48ga).

## Footnotes

1 Of course, it is possible that participants experience intrinsic reward from their motivation to do well in the task.

2 Additionally, such studies may also distinguish between reward cues at the beginning of a trial (i.e., reward expectation) and the actual reward presented at the end of the trial (i.e., outcome). RPE related to reward cues is the difference between potential reward on that trial and average reward across trials (Bunzeck et al., 2010; Jang et al., 2019; Rouhani & Niv, 2021). In this manuscript, we focus on RPE related to the outcome as most relevant to one-shot learning (i.e., the difference between expected and actual outcomes).

3 All self-paced tasks were capped at 60 s per response to preclude the possibility of extended breaks mid-task. Having null responses on more than five consecutive trials automatically terminated the experiment, and participants were compensated for the time spent on the experiment until that point.

4 There were not enough datapoints to estimate interactions with Collection in the polynomial model.

5 Note that the definition of PE actually relates to the interaction between the factors of Reward and #Options.

## Code availability

Code is available on OSF (https://osf.io/b48ga).

## Declarations

### Open practices

All data and code are available on OSF (https://osf.io/b48ga), and all experiments were preregistered: Experiment 1A at https://osf.io/eubzf, Experiment 1B at https://osf.io/8wtsn, and Experiment 2 at https://osf.io/nz295.
