# Supplementary material for: Does signed prediction error drive declarative memory? Evidence from variable choice paradigms
Source: Mem Cognit. 2026 Mar 17;54(6):2212–27. doi: 10.3758/s13421-026-01864-8 (PMC13461684; doi:10.3758/s13421-026-01864-8)
Supplement: Supplementary file 1 [file 13421_2026_1864_MOESM1_ESM.docx]

Supplementary Materials for

Does Signed Prediction Error drive Declarative Memory? Evidence from Variable Choice Paradigms

Kshipra Gurunandan, Andrea Greve, Ella Wilmot & Richard N Henson

# 1. Multiple-trace model fits for experimental subsets of data

Figure S1 illustrates the model fits for each of the experimental subsets which were combined in Figure 4 of the main manuscript.

Figure S1. Multiple-trace model (in blue) plotted over data from five experimental subsets. Rewarded trials are plotted in green, unrewarded trials in red. Error bars are 95% confidence intervals of the empirical data means.

Table 3 in the main paper shows the single best parameter estimates when fitting the average data across participants. The model can also be fit to individual participants’ data, as shown in Table S1 below. Note that nonlinear fitting means that the average parameter value after fitting each participant will not necessarily match the single parameter value after fitting the average data across participants. The results for the *Target* and *Preference* traces in Table S1 are similar to those in Table 3, but the results for the *Choice* trace are different, with the mean now being above zero for all datasets (whereas this parameter was not needed for most datasets in Table 3). This reflects variation across individual participant estimates, though most participants had zero values for the *Choice* trace (median = 0) and it does not affect any of the conclusions in the paper.

Table S1. Mean model parameter values for each experimental subset (original VCP), with standard deviation in brackets.

| **Experiment** | **Subset** | **Model Parameters (triple trace)** | | |
| --- | --- | --- | --- | --- |
|  |  | *P(target)* | *P(preference)* | *P(choice)* |
| De Loof et al., 2018 | In-person | 0.54 (0.19) | 0.22 (0.28) | 0.26 (0.34) |
|  | In-person (next day) | 0.31 (0.17) | 0.28 (0.30) | 0.15 (0.16) |
| Experiment 1A | In-person | 0.55 (0.21) | 0.26 (0.29) | 0.08 (0.15) |
|  | Online | 0.40 (0.18) | 0.16 (0.26) | 0.08 (0.10) |
| Experiment 1B | Online | 0.40 (0.21) | 0.17 (0.22) | 0.11 (0.23) |

Similarly, we have illustrated below the fits of simplest to more complex models for the data from Ergo et al. (2021) in Figure S2, and Table S2 shows the average parameter values after fitting each participant.

Table S2. Mean model parameter values for each model fitted to data from Ergo et al. (2021), with standard deviation in brackets.

| **Model** | **Agency main effect** | **Model Parameters** | | |
| --- | --- | --- | --- | --- |
|  | *NoAgencyCost* | *P(target)* | *P(preference)* | *P(choice)* |
| Dual Trace | - | 0.45 (0.18) | 0.36 (0.28) | - |
|  | 0.21 (0.22) | 0.51 (0.20) | 0.39 (0.33) | - |
| Triple Trace | - | 0.44 (0.18) | 0.26 (0.28) | 0.13 (0.16) |
|  | 0.24 (0.24) | 0.50 (0.20) | 0.29 (0.31) | 0.19 (0.27) |

Figure S2. Illustrated fits for four multiple-trace models (in blue) from simplest to more complex, plotted over empirical data from Ergo et al. (2021). Rewarded trials are plotted in green, unrewarded trials in red. Error bars are 95% confidence intervals of the empirical data means.

Finally, in Figure S3, we overlay linear fits of the SPE and multiple-trace models for the data from Ergo et al. (2021) to illustrate the similarity in their predictions. Though Ergo et al. reported a Bayes Factor that supported no difference in slopes, linear mixed effects SPE models show numerical differences in the slopes (β = 0.26 in the “agency” condition and β = 0.16 in the “no agency” condition) consistent with the predictions of the multiple trace model.

Figure S3. Illustrated linear fits for the SPE account (black) and the multiple trace account (blue). Rewarded trials are plotted in green, unrewarded trials in red. Error bars show 95% confidence intervals of the empirical data means.
